# Editorial: Translating nanomedicines for anti-cancer treatment

**DOI:** 10.3389/fphar.2023.1236981

**Published:** 2023-09-08

**Authors:** Karthik Siram, Hardik Hastibhai Amin, Nileshkumar Meghani, Habibur Rahman, Raghuram Kandimalla, Shivendu Ranjan

**Affiliations:** ^1^ Center for Translational Medicine, Department of Biomedical and Pharmaceutical Sciences, Skaggs School of Pharmacy, University of Montana, Missoula, MT, United States; ^2^ Inimmune Corp., Missoula, MT, United States; ^3^ PSG College of Pharmacy, Coimbatore, Tamil Nadu, India; ^4^ Brown Cancer Center, University of Louisville, Louisville, KY, United States; ^5^ School of Nano Science and Technology, Indian Institute of Technology Kharagpur, Kharagpur, West Bengal, India

**Keywords:** nanomedicine, cancer, cancer immunology, cancer vaccine, cancer immunomodulator, imaging and diagnosis

Nanomedicine is a rapidly emerging field that holds great promise for the treatment of cancer. Nanomedicine has the potential to completely alter the way cancer is treated since it can precisely target cancer cells while causing the least amount of damage to healthy organs. But creating efficient nanomedicines for cancer therapy is a difficult challenge that necessitates a multidisciplinary approach. The translation of preclinical discoveries to clinical trials and, finally, to the clinic, is one of the major obstacles in the development of effective nanomedicines for cancer therapy. Preclinical research has shown that a variety of nanomedicines, including liposomes, exosomes, polymeric nanoparticles, and dendrimers, have the ability to carry anticancer therapeutics directly to tumour cells, increasing their effectiveness and lowering their toxicity. But bringing these discoveries to the clinic has proven to be a significant challenge. (Maddinedi et al., 2015; Balaji et al., 2017; Dasgupta et al., 2018; Kadiyala et al., 2018; Meghani et al., 2018; Ranjan et al., 2020; Moholkar et al., 2023).

There are several reasons for this difficulty in translation. The first issue is that it is challenging to precisely forecast how nanomedicines would behave *in vivo* due to their complexity and interactions with biological systems. Second, regulatory requirements for the development and approval of nanomedicines are not yet well-defined, leading to uncertainty and delays in the regulatory process. Third, there is a lack of standardization in the characterization of nanomedicines, which makes it difficult to compare findings from various investigations.

A collaborative and multidisciplinary strategy must be established to the creation of nanomedicines for the treatment of cancer to overcome these obstacles. This strategy must engage specialists in a variety of domains, including clinical oncology, pharmacology, toxicology, and nanotechnology. To guarantee that the development of nanomedicines is in compliance with regulatory standards and can be applied in clinical practice, collaboration between academia, industry, and regulatory bodies is also essential. Another crucial issue that has to be addressed is standardization in the characterisation of nanomedicines. A consistent methodology will make it possible to compare the findings of various investigations and make it easier to create consensus criteria for the creation and characterisation of nanomedicines. This will improve the regulatory approval procedure in addition to improving the quality of preclinical and clinical investigations.

This Research Topic focuses on new developments in cancer treatment using nanomedicine that may find use in clinical settings. Zhao et al. investigated numerous new developments in the pancreatic cancer treatment using nano drug delivery systems (NDDS) and presented in a comprehensive review article. On the other hand, Kumar et al. reported on a variety of synthetic and natural nanomaterials that were applied to the delivery of chemotherapy drugs for the treatment of breast cancer. Nanoscale core/shell structures known as polymeric micelles are created by amphiphilic block copolymers. In recent times, several pre-clinical investigations were conducted by employing the polymeric micelles to deliver chemotherapeutic drugs with solubility issues. Guo et al. showed that HT001 (clinical stage docetaxel micelle), which aimed to increase the water solubility and safety profile, has anti-tumour and ascites-inhibitory properties. HT001 has antitumor efficacy against ovarian, lung, and breast solid tumours and lessens ascites brought on by liver cancer cells. Tumours create a unique microenvironment that can suppress immune responses and promote tumour growth. Immune evasion mechanisms employed by cancer cells include the expression of immune checkpoint molecules (e.g., PD-L1), recruitment of immunosuppressive cells (e.g., regulatory T cells, myeloid-derived suppressor cells), and secretion of immunosuppressive cytokines. Understanding these mechanisms is essential for developing strategies to overcome immune evasion and enhance anti-tumour immunity (Joyce and Fearon, 2015; Chen and Mellman, 2017). Brown et al. presented a brief research report on nano formulation of monoamine oxidase inhibitors (MAOIs) that were used as immune checkpoint blockade therapy.

In recent years, more effective drug delivery to the targeted tissues has been achieved using ultrasonic (US) waves. Heat produced by US waves aids in the drug’s dispersion from the nanocarriers at the desired location and initiates sonoporation of the delivered therapeutics into the cancer cells. Wang et al. addressed the potentiality of US waves for the delivery of chemotherapeutics for the ovarian cancer treatment. Another novel method used recently to treat hypervascularized tumors is drug-eluting beads trans arterial chemoembolization (DEB-TACE). This method involves injecting chemo drug-loaded beads into the arteries that provide blood to tumours, which further embolizes the artery and induces an ischemia situation in the tumour. This technique is widely used for the treatment of hepatic tumours. Bi et al. employed transarterial chemoembolization with oxaliplatin-loaded drug-eluting beads in a clinical study to treat advanced lung cancer, and they found that it was efficient and safe for all the patients examined. Gene therapy is another therapeutic approach that has been extensively employed to deliver therapeutic nucleic acids to cancer cells, such as small interfering RNA (siRNA), plasmid DNA (pDNA), and messenger RNA (mRNA), for efficient treatment (Sridharan and Gogtay, 2016; Munagala et al., 2021). It can be difficult to transport these therapeutic nucleic acids to cancer cells, thus researchers are exploring for effective delivery vectors. Wu et al. reported a nano system that can deliver CRISPR/Cas9-3NLS/sgHMGA2 for the HMGA2 gene editing and aid in gastric cancer treatment.

In conclusion, the development of effective nanomedicines for cancer treatment holds great promise but requires a collaborative and interdisciplinary approach. Standardization in the characterization of nanomedicines is also critical for the translation of preclinical findings to clinical trials and ultimately to the clinic. With continued research and collaboration, we can overcome these challenges and realize the full potential of nanomedicines for the treatment of cancer. The use of nanotechnology in cancer immunology holds great promise for improving cancer treatment outcomes. However, more research is needed to fully understand the safety and efficacy of nanoparticle-based therapies.

## References

[B1] Balaji S. Mandal B. K. Ranjan S. Dasgupta N. Chidambaram R. (2017). Nano-zirconia – evaluation of its antioxidant and anticancer activity. J. Photochem Photobiol. B Biol. 170, 125–133. 10.1016/j.jphotobiol.2017.04.004 28431297

[B2] Chen D. S. Mellman I. (2017). Elements of cancer immunity and the cancer-immune set point. Nature 541, 321–330. 10.1038/nature21349 28102259

[B3] Dasgupta N. Ranjan S. Mishra D. Ramalingam C. (2018). Thermal Co-reduction engineered silver nanoparticles induce oxidative cell damage in human colon cancer cells through inhibition of reduced glutathione and induction of mitochondria-involved apoptosis. Chem. Biol. Interact. 295, 109–118. 10.1016/j.cbi.2018.07.028 30056045

[B4] Joyce J. A. Fearon D. T. (2015). T cell exclusion, immune privilege, and the tumor microenvironment. Science 348 (80), 74–80. 10.1126/science.aaa6204 25838376

[B5] Kadiyala N. K. Mandal B. K. Ranjan S. Dasgupta N. (2018). Bioinspired gold nanoparticles decorated reduced graphene oxide nanocomposite using Syzygium cumini seed extract: Evaluation of its biological applications. Mater Sci. Eng. C 93, 191–205. 10.1016/j.msec.2018.07.075 30274051

[B6] Maddinedi S. B. Mandal B. K. Ranjan S. Dasgupta N. (2015). Diastase assisted green synthesis of size-controllable gold nanoparticles. RSC Adv. 5, 26727–26733. 10.1039/c5ra03117f

[B7] Meghani N. Patel P. Kansara K. Ranjan S. Dasgupta N. Ramalingam C. (2018). Formulation of vitamin D encapsulated cinnamon oil nanoemulsion: Its potential anti-cancerous activity in human alveolar carcinoma cells. Colloids Surfaces B Biointerfaces 166, 349–357. 10.1016/j.colsurfb.2018.03.041 29631227

[B8] Moholkar D. N. Kandimalla R. Gupta R. C. Aqil F. (2023). Advances in lipid-based carriers for cancer therapeutics: Liposomes, exosomes and hybrid exosomes. Cancer Lett. 565, 216220. 10.1016/j.canlet.2023.216220 37209944 PMC10325927

[B9] Munagala R. Aqil F. Jeyabalan J. Kandimalla R. Wallen M. Tyagi N. (2021). Exosome-mediated delivery of RNA and DNA for gene therapy. Cancer Lett. 505, 58–72. 10.1016/j.canlet.2021.02.011 33610731 PMC8005491

[B10] Ranjan S. Dasgupta N. Mishra D. Ramalingam C. (2020). Involvement of bcl-2 activation and G1 cell cycle arrest in colon cancer cells induced by titanium dioxide nanoparticles synthesized by microwave-assisted hybrid approach. Front. Bioeng. Biotechnol. 8, 606. 10.3389/fbioe.2020.00606 32760701 PMC7373722

[B11] Sridharan K. Gogtay N. J. (2016). Therapeutic nucleic acids: Current clinical status. Br. J. Clin. Pharmacol. 82, 659–672. 10.1111/bcp.12987 27111518 PMC5338117

